# Evaluate the stability of the vehicle when using the active suspension system with a hydraulic actuator controlled by the OSMC algorithm

**DOI:** 10.1038/s41598-022-24069-w

**Published:** 2022-11-12

**Authors:** Duc Ngoc Nguyen, Tuan Anh Nguyen

**Affiliations:** Thuyloi University, Hanoi, Vietnam

**Keywords:** Mechanical engineering, Electrical and electronic engineering

## Abstract

The ride comfort is controlled by the suspension system. In this article, an active suspension system is used to control vehicle vibration. Vehicle oscillations are simulated by a quarter-dynamic model with five state variables. This model includes the influence of the hydraulic actuator in the form of linear differential equations. This is a completely novel model. Besides, the OSMC algorithm is proposed to control the operation of the active suspension system. The controller parameters are optimized by the in-loop algorithm. According to the results of the study, under normal oscillation situations, the maximum and average values of the sprung mass were significantly reduced when the OSMC algorithm was applied. In dangerous situations, the wheel is completely separated from the road surface if the vehicle uses only the passive suspension system or active suspension system with a conventional linear control algorithm. In contrast, the interaction between the wheel and the road surface is always guaranteed when the OSMC algorithm is used to control the operation of the active suspension system. The efficiency that this algorithm brings is very high.

## Introduction

The suspension system is one of the most important systems in a vehicle. Over the years, the suspension system has been more and more perfected and developed. The suspension system is used to ensure that the vehicle’s vibrations are within the allowable limits. The structure of the passive suspension system is quite simple, including a coil spring (or leaf spring), a damper, and a lever arm. Besides, the stabilizer bar is also considered part of the suspension system^1^. The suspension system is located between the axle and the body of the vehicle. It divides the vehicle into two separate parts, including the sprung mass and the unsprung mass^2^. When the vehicle’s body vibrates, the internal forces generated by the suspension system act on both parts of the mass to control the vibrations. The stiffness of these components plays an important role in ensuring the vehicle’s smoothness when traveling on the road. For a passive suspension system, it is not possible to change the stiffness of the spring or damper. Therefore, smoothness will not be guaranteed in many cases. As a result, several automatically controlled suspension systems have been used to replace conventional mechanical suspensions. In Ref.^3^, Zepeng et al. introduced the use of air suspension on the vehicle. This suspension system uses an air balloon (air spring) to replace the metal spring. As air is supplied or withdrawn from the balloon, the pressure inside changes. This causes the stiffness of the air spring to change continuously in response to the driving conditions of the vehicle^4^. Besides, changing the stiffness of the damper is also a concern. In Ref.^5^, Khedkar et al. presented an electronic damping model with an average response. The circulation rate of the liquid inside the damper depends on the arrangement of the iron particles inside. When the vehicle’s body vibrates, an electric current signal is applied to the inside of the damper. A magnetic field will appear that changes the arrangement of small metal particles. Therefore, the damping stiffness can be changed^6^. Because the response of the system is still incomplete, the suspension system is therefore called semi-active. To improve the efficiency of the suspension system, an active suspension system was used. Each wheel has a hydraulic actuator as part of the active suspension^7^. This mechanism works based on the opening and closing of servo valves. The process of opening and closing the valve gates is controlled by the current signal of the controller^8^. According to Nguyen, the hydraulic actuator will generate force to act on both the sprung and unsprung masses. This force is used to reduce the vibrations of the vehicle. From there, the smoothness of the vehicle will be significantly improved^9^.

Several control algorithms for the active suspension system have been introduced in recent times. Anh used the PID control algorithm for this system in Ref.^10^. The PID algorithm is suitable for SISO linear systems. Abdullah et al. presented the MOPID model for the active suspension system in Ref.^11^. In this model, three controllers are used. These three controllers will control the KP, KI, and KD coefficients of the system, respectively. The parameters of the PID controller can be determined by the Ziegler–Nichols method. However, its accuracy will not be high^12,13^. Besides, these parameters can be determined more optimally thanks to some intelligent algorithms, such as GSA, PSO, etc.^14,15^. The values of these parameters can also be changed continuously thanks to the use of a Fuzzy-PID hybrid controller. If the fuzzy rule is properly constructed, this change is very positive^16–18^. If the system has many objects to be considered, that is, a MIMO system, the LQR control algorithm is the suitable alternative^19^. According to Maurya and Bhangal, the LQR algorithm uses state matrices instead of the usual system of differential equations^20^. According to Rodriguez-Guevara et al., the LQR algorithm is designed based on the optimization of the cost function^21^. At that time, the efficiency of the system can reach a high level. To limit the influence of noise, a Gaussian filter is often combined with this algorithm^22–24^.

In practice, vehicle oscillations are often random. These are nonlinear oscillations. Conventional linear controllers cannot guarantee stable performance in many cases. In Ref.^25^, Bai and Guo proposed the use of the SMC algorithm for the active suspension system. For this algorithm, it is necessary to determine the state variables of the oscillatory state matrix^26^. If hydraulic actuators are considered, the number of possible state variables is 5 (for a quarter-dynamics model), or 10 (for a half-dynamics model)^27,28^. This will make the process of derivation of state variables extremely difficult. Therefore, Nguyen performed the process of linearizing the hydraulic actuator to be used for the SMC algorithm with 5 state variables^29^. The SMC algorithm can also be combined with the PSO algorithm to improve efficiency when choosing controller parameters^30^. Some oscillations change continuously and are not fixed in any state. Therefore, the Fuzzy algorithm was proposed to determine these states. The Fuzzy algorithm can be used for the suspension system, stabilizer bar, and many other control systems^31,32^. This algorithm can respond to the continuous change of the input signal, which has been shown in the article by Mustafa et al.^33^. Fuzzy control laws can be determined based on the experience of the designer. Besides, the Fuzzy algorithm can be easily combined with many other algorithms^34–36^. In general, the efficiency of nonlinear and intelligent control methods is very high^37–40^. In addition, some optimization methods used to select controller parameters have also been applied in some papers^41–43^. The results brought from these methods are positive.

## Methods

### Model of the vehicle dynamics

**Figure 1:**
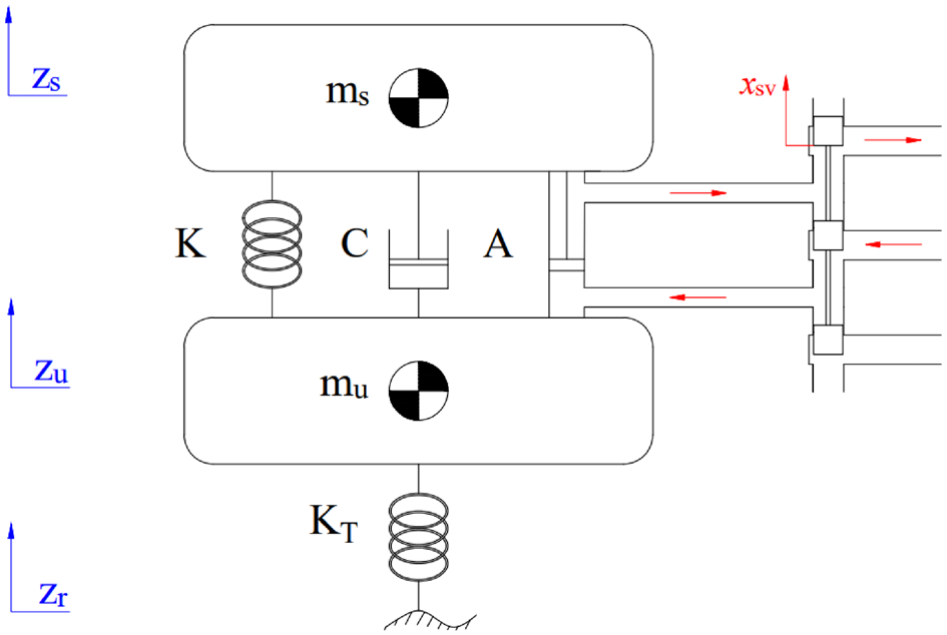
The quarter-dynamics model.

There are many dynamic models used to simulate vehicle vibrations. For control problems, a quarter-dynamics model is often used. This model includes two masses: the sprung mass *m*_*s*_, and the unsprung mass, *m*_*u*_ (Fig. 1). The system of equations describing the vibration of the vehicle is written in the following form:

1 $$ F_{is} - F_{K} - F_{C} - F_{A} = 0, $$

2 $$ F_{iu} - F_{KT} + F_{K} + F_{C} + F_{A} = 0, $$

where

3 $$ F_{is} = m_{s} \ddot{z}_{s} , $$

4 $$ F_{iu} = m_{u} \ddot{z}_{u} , $$

5 $$ F_{K} = K\left( {z_{u} - z_{s} } \right), $$

6 $$ F_{C} = C\left( {\dot{z}_{u} - \dot{z}_{s} } \right), $$

7 $$ F_{KT} = K_{T} \left( {z_{r} - z_{u} } \right). $$

Determining the impact force, *F*_*A*_ is quite difficult. This value depends on a complex nonlinear function. A linear function is used to simplify the determination of the impact force *F*_*A*_^29^.

8 $$ F_{A} = \kappa_{1} \int {\left( {i\left( t \right) - \kappa_{2} F_{A} } \right)dt} + \kappa_{3} z_{sus} . $$

### Nonlinear control theory

Assume that the nonlinear function has only one value and that there is a derivative at each level about the equilibrium point, *x*_*c*_. The nonlinear function has the form:

9 $$ \dot{x}_{i} = f_{i} \left( x \right). $$

The deviation can be represented by Eq. (10). This equation is an expansion of the Taylor series.

10 $$ \Delta x_{i} = \frac{{\partial f_{i} }}{{\partial x_{1} }}\left| {_{{x = x_{c} }} } \right.\Delta x_{1} + \frac{{\partial f_{i} }}{{\partial x_{2} }}\left| {_{{x = x_{c} }} } \right.\Delta x_{2} + \frac{{\partial f_{i} }}{{\partial x_{3} }}\left| {_{{x = x_{c} }} } \right.\Delta x_{3} + \cdots = \sum\limits_{i = 1}^{n} {\sum\limits_{j = 1}^{n} {\frac{{\partial f_{i} }}{{\partial x_{j} }}\left| {_{{x = x_{c} }} } \right.\Delta x_{j} } } . $$

Equation (10) can be rewritten as:

11 $$ \Delta x_{i} = P\Delta x_{j} , $$

where

12 $$ P = \left| {\frac{{\partial f_{m} }}{{\partial x_{n} }}} \right|_{m \times n} . $$

The linear approximation characteristic equation of the system is:

13 $$ det\left( {sI - P} \right) = 0, $$

where *I* is the unit matrix.

According to Lyapunov theory, if the solution of Eq. (13) has a non-zero real part, then approximately linear characteristic equations always give the correct answer regarding the stability of the nonlinear system. If all the solutions to Eq. (13) have a negative real part, then the nonlinear system will be stable in a narrow range (14).

14 $$ ReS_{i} < 0. $$

Conversely, if only one of the solutions to Eq. (13) has a positive real part, then the system is not stable. This is the view of Lyapunov’s first method when considering the stability of nonlinear systems. For this method, the disturbed motion has been rewritten as an approximate linear differential equation. The accuracy of the system after linearization is not high. To improve this problem, the second method of Lyapunov was proposed. According to the second method, some integral stages of the system can be omitted.

A nonlinear system can be described by a set consisting of many first-order linear differential equations.

15 $$ \dot{x}_{i} = f\left( {x_{i} ,t} \right). $$

The system of Eq. (15) can be rewritten as a matrix of states:

16 $$ \left[ {\begin{array}{*{20}c} {\dot{x}_{1} } \\ {\dot{x}_{2} } \\ \cdots \\ {\dot{x}_{n} } \\ \end{array} } \right] = \left[ {\begin{array}{*{20}c} {f_{1} \left( {x_{i} ,t} \right)} \\ {f_{2} \left( {x_{i} ,t} \right)} \\ \cdots \\ {f_{n} \left( {x_{i} ,t} \right)} \\ \end{array} } \right]. $$

#### Lyapunov’s theorem

If a positive definite function is found $V\left( x \right) = V\left( {x_{1} ,x_{2} ,...,x_{n} } \right)$ such that its derivative according to the differential equation of the disturbed motion $\dot{x}_{1} = f\left( {x_{1} ,x_{2} ,...,x_{n} } \right)$ is negatively defined, the undisturbed motion will be asymptotically stable.

The quadratic form of the function *V(x)*:

17 $$ V\left( x \right) = \frac{1}{2}\sum\limits_{i = 1}^{n} {\sum\limits_{j = 1}^{n} {q_{ij} x_{i} x_{j} } } . $$

#### Sylvester’s theorem

The quadratic form of the function *V(x)* is positive definite if and only if all the principal diagonal determinants of the symmetric matrix *Q* must be positive.

18 $$ Q = \left[ {q_{ij} } \right]_{i \times j} , $$

19 $$ V\left( x \right) > 0 \Leftrightarrow \Delta_{k} = \left| {q_{ij} } \right|_{i \times j} > 0. $$

Consider the nonlinear object of degree *n* described by Eq. (20):

20 $$ \left\{ \begin{array}{*{20}l} \dot{x} = f\left( x \right) + g\left( x \right)i\left( t \right) \hfill \\ y = h\left( x \right) \hfill \\ \end{array} \right., $$

where *x* is the state vector of the system, *y* is the output signal.

Take the derivative of the output signal *n* times:

21 $$ y^{\left( n \right)} = a\left( x \right) + b\left( x \right)i\left( t \right) = L_{f}^{n} h\left( x \right) + L_{g} L_{f}^{n - 1} h\left( x \right)i\left( t \right), $$

with

22 $$ L_{f}^{k} = \frac{{\partial L_{f}^{k - 1} }}{\partial \left( x \right)}f\left( x \right), $$

23 $$ L_{g} = \frac{\partial g\left( x \right)}{{\partial \left( x \right)}}. $$

Substitute (22) and (23) into (21):

24 $$ \begin{aligned} y^{\left( n \right)} = & a\left( x \right) + b\left( x \right)i\left( t \right) = \frac{{\partial L_{f}^{n - 1} }}{\partial \left( x \right)}f\left( x \right)h\left( x \right) + \frac{{\partial L_{f}^{n - 1} }}{\partial \left( x \right)}g\left( x \right)h\left( x \right)i\left( t \right) \\ = & \frac{{\partial L_{f}^{n - 1} }}{\partial \left( x \right)}h\left( x \right)\left( {f\left( x \right) + g\left( x \right)i\left( t \right)} \right). \\ \end{aligned} $$

Let *e(t)* be the error between the setpoint signal and the output signal:

25 $$ e\left( t \right) = y_{s} \left( t \right) - y\left( t \right). $$

The sliding surface is designed so that the signal error is minimized.

26 $$ \sigma \left( t \right) = e^{{\left( {n - 1} \right)}} + p_{1} e^{{\left( {n - 2} \right)}} + \cdots + p_{n - 2} \dot{e} + p_{n - 1} e. $$

Coefficients *p*_*i*_ are chosen so that the polynomial *γ(s)* is a Hurwitz polynomial.

27 $$ \gamma \left( s \right) = s^{n - 1} + p_{1} s^{n - 2} + \cdots + p{}_{n - 2}s + p_{n - 1} . $$

Choose the Lyapunov function:

28 $$ V = \frac{1}{2}\sigma^{2} . $$

For the system to be stable, the derivative of *V(x)* needs to be negative, i.e.:

29 $$ \dot{V} = \sigma \dot{\sigma } < 0. $$

Based on Lyapunov stability theory, the SMC algorithm can be established.

### OSMC controller design

The recommended OSMC algorithm is used to control the operation of the active suspension system. Combining equations from (1) to (8), we get:

30 $$ m_{s} \ddot{z}_{s} = - Kz_{s} - C\dot{z}_{s} + Kz_{u} + C\dot{z}_{u} + F_{A} , $$

31 $$ m_{u} \ddot{z}_{u} = Kz_{s} + C\dot{z}_{s} - \left( {K + K_{T} } \right)z_{u} - C\dot{z}_{u} - F_{A} + K_{T} z_{r} . $$

For the quarter-dynamics model, the inertia force Fi of the sprung mass and the unsprung mass is considered to be proportional. This means that:

32 $$ \ddot{z}_{s} = \chi \left( {z_{r} - z_{u} } \right), $$

where *χ* is the proportional factor.

Let:

33 $$ \begin{gathered} x_{1} = z_{s} \hfill \\ x_{2} = \dot{z}_{s} \hfill \\ x_{3} = z{}_{u} \hfill \\ x_{4} = \dot{z}_{u} \hfill \\ x_{5} = F_{A} . \hfill \\ \end{gathered} $$

Taking the derivative of state variables:

34 $$ \begin{array}{*{20}l} \dot{x}_{1} = x_{2} \hfill \\ \dot{x}_{2} = \frac{1}{{m_{s} }}\left( { - Kx_{1} - Cx_{2} + Kx_{3} + Cx_{4} + x_{5} } \right) \hfill \\ \dot{x}_{3} = x_{4} \hfill \\ \dot{x}_{4} = \frac{1}{{m_{u} }}\left( {Kx_{1} + Cx_{2} - \left( {K + K_{T} } \right)x_{3} - Cx_{4} - x_{5} } \right) \hfill \\ \dot{x}_{5} = - \kappa_{3} x_{2} + \kappa_{3} x_{4} - \kappa_{2} x_{5} + \kappa_{1} i\left( t \right). \hfill \\ \end{array} $$

In this article, the value of displacement of the sprung mass is considered as the output of the controller. Therefore:

35 $$ y = x_{1} . $$

Take the *n*-order derivative of the output signal. Where *n* is the number of state variables:

36 $$ \dot{y} = x_{2} , $$

37 $$ \ddot{y} = - \frac{{K_{T} }}{{\chi m_{s} }}x_{3} , $$

38 $$ y^{\left( 3 \right)} = - \frac{{K_{T} }}{{\chi m_{s} }}x_{4} , $$

39 $$ y^{\left( 4 \right)} = - \frac{{K_{T} }}{{\lambda m_{s} m_{u} }}\left( {Kx_{1} + Cx_{2} - \left( {K + K_{T} } \right)x_{3} - Cx_{4} - x_{5} } \right), $$

40 $$ y^{\left( 5 \right)} = \frac{{K_{T} }}{{\chi m_{s} m_{u} }}\left( \begin{gathered} \left( {\frac{KC}{{m_{s} }} + \frac{KC}{{m_{u} }}} \right)x_{1} + \left( {\frac{{C^{2} }}{{m_{s} }} + \frac{{C^{2} }}{{m_{u} }} - K - \kappa_{3} } \right)x_{2} \hfill \\ + \left( { - \frac{KC}{{m_{s} }} - \frac{{\left( {K + K_{T} } \right)C}}{{m_{u} }}} \right)x_{3} \hfill \\ + \left( { - \frac{{C^{2} }}{{m_{s} }} - \frac{{C^{2} }}{{m_{u} }} + \left( {K + K_{T} } \right) + \kappa_{3} } \right)x_{4} \hfill \\ + \left( { - \frac{C}{{m_{s} }} - \frac{C}{{m_{u} }} - \kappa_{2} } \right)x_{5} \hfill \\ \end{gathered} \right) + \frac{{K_{T} \kappa_{1} }}{{\chi m_{s} m_{u} }}i\left( t \right). $$

Let:

41 $$ \begin{array}{*{20}l} b_{1} = \frac{{K_{T} }}{{\chi m_{s} m_{u} }} \hfill \\ b_{2} = \frac{{K_{T} \kappa_{1} }}{{\chi m_{s} m_{u} }} \hfill \\ a_{1} = \frac{KC}{{m_{s} }} + \frac{KC}{{m_{u} }} \hfill \\ a_{2} = \frac{{C^{2} }}{{m_{s} }} + \frac{{C^{2} }}{{m_{u} }} - K - \kappa_{3} \hfill \\ a_{3} = - \frac{KC}{{m_{s} }} - \frac{{\left( {K + K_{T} } \right)C}}{{m_{u} }} \hfill \\ a_{4} = - \frac{{C^{2} }}{{m_{s} }} - \frac{{C^{2} }}{{m_{u} }} + \left( {K + K_{T} } \right) + \kappa_{3} \hfill \\ a_{5} = - \frac{C}{{m_{s} }} - \frac{C}{{m_{u} }} - \kappa_{2} . \hfill \\ \end{array} $$

Equation (40) can be rewritten as:

42 $$ y^{\left( 5 \right)} = b_{1} \sum\limits_{i = 1}^{5} {a_{i} x_{i} } + b_{2} i\left( t \right) $$

The sliding surface of the controller is taken according to the error signal with derivative order *n − *1:

43 $$ \sigma = e^{\left( 4 \right)} + p_{1} e^{\left( 3 \right)} + p_{2} \ddot{e} + p_{3} \dot{e} + p_{4} e = \sum\limits_{i = 0}^{4} {p_{i} e^{{\left( {4 - i} \right)}} } . $$

With *p*_*i*_ being the coefficients of the polynomial (27).

Combining Eqs. (20), (25), (42), and (43), the output signal of the controller has the form:

44 $$ i\left( t \right) = b_{3} \left( {y_{s}^{\left( 5 \right)} - \sum\limits_{i = 1}^{5} {a_{i} x_{i} + \sum\limits_{i = 1}^{4} {p_{i} e^{{\left( {4 - i} \right)}} } + Rsgn\left( {\sum\limits_{i = 0}^{4} {p_{i} e^{{\left( {4 - i} \right)}} } } \right)} } \right), $$

where

45 $$ b_{3} = \frac{{\chi m_{s} m_{u} }}{{K_{T} \kappa_{1} }}. $$

**Figure 2:**
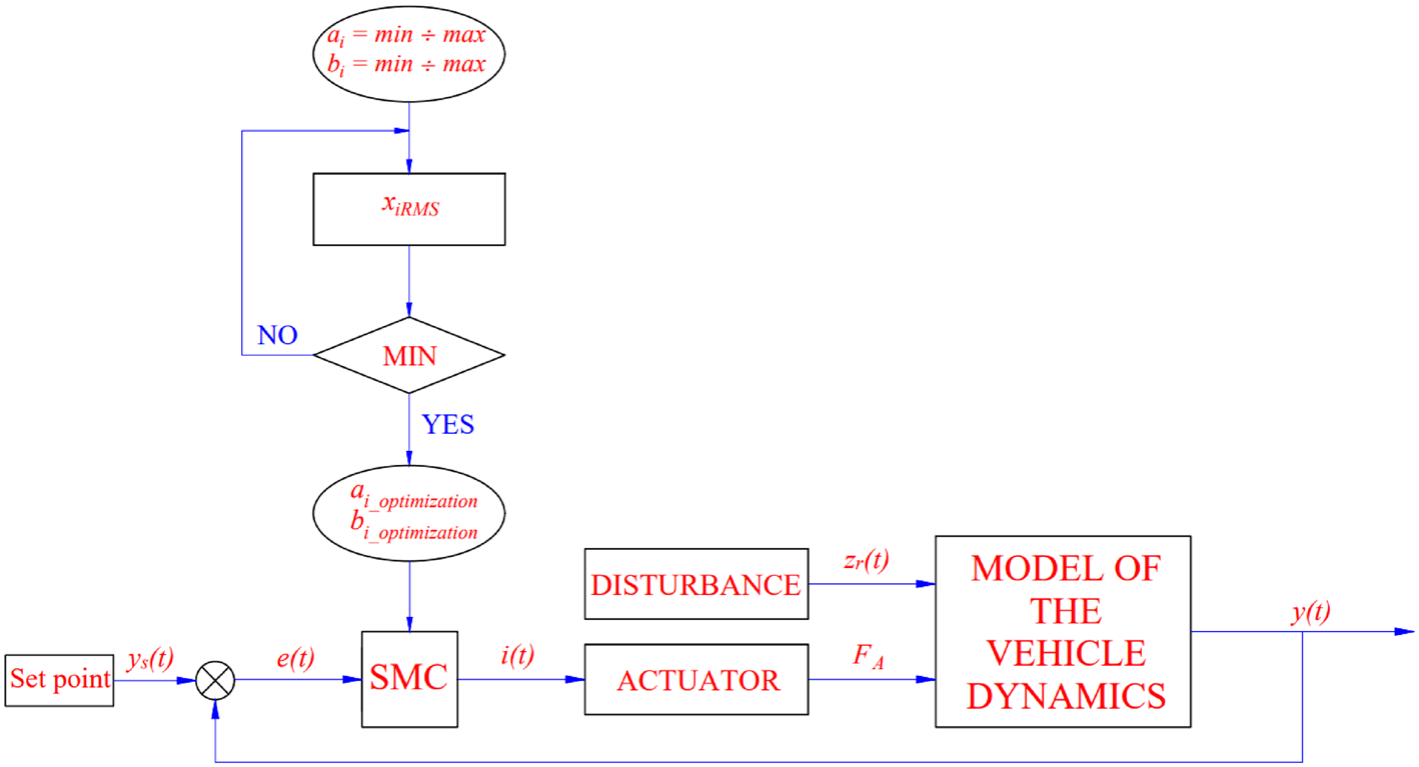
OSMC algorithm.

The parameters *a*_*i*_ and *p*_*i*_ of Eq. (44) are determined through the in-loop optimization algorithm. Their values will be used in turn to find the most optimal value that minimizes the vehicle’s vibrations. The general diagram of the OSMC algorithm is given as shown in Fig. 2.

## Results

### Investigation cases

**Figure 3:**
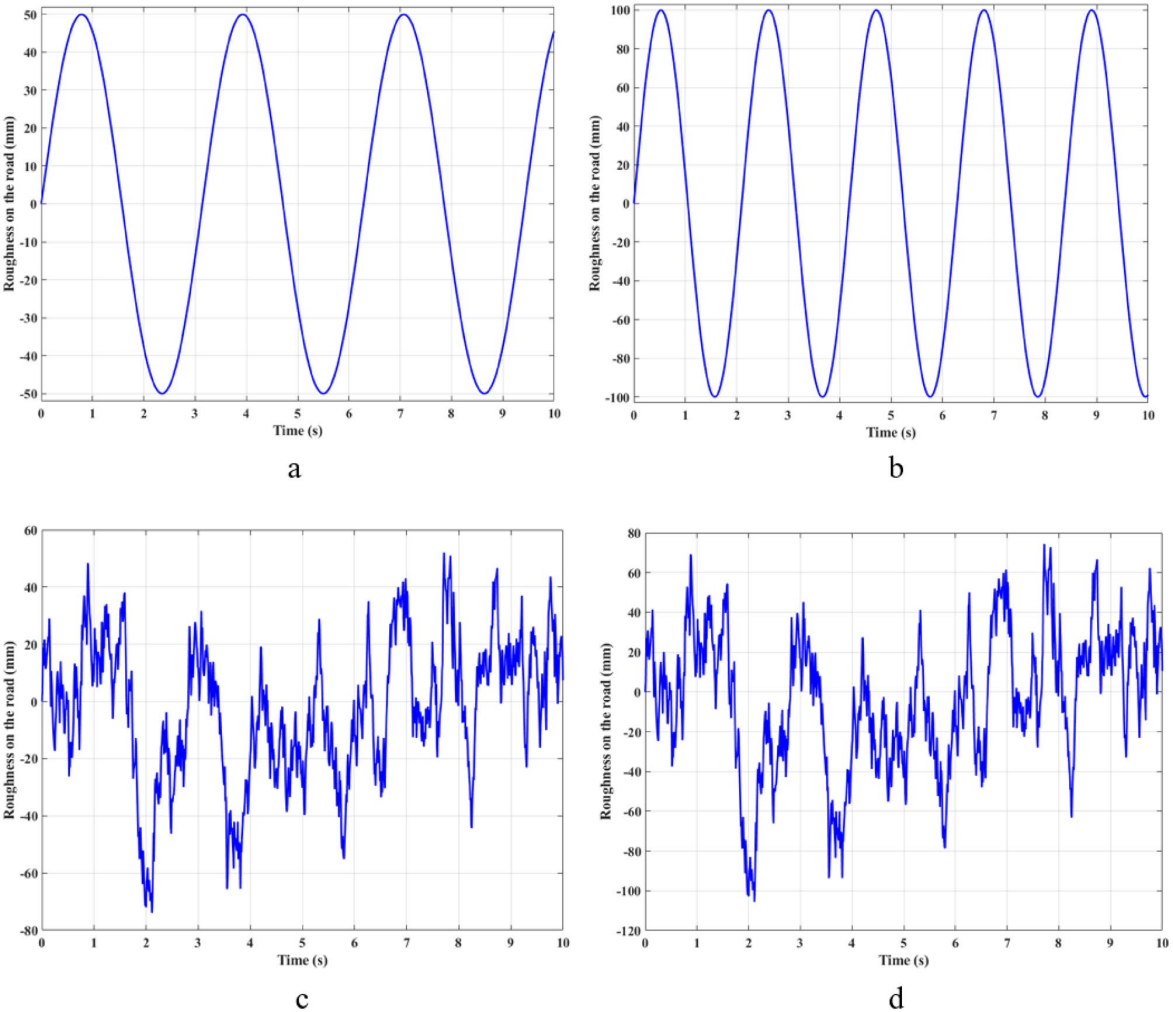
Roughness on the road ((**a**) Case 1; (**b**) Case 2; (**c**) Case 3; (**d**) Case 4).

This study only conducted simulations. The simulation is performed by the MATLAB-Simulink software. There are four types of excitations from the pavement that are used to conduct the vehicle vibration calculation. These four forms of stimulation correspond to the corresponding four cases (Fig. 3). These are the main pavement excitation types commonly used in simulation problems. As for the first two types (Fig. 3a,b), they are sine-shaped. This is the usual form of circulatory stimulation. The values of amplitude and frequency in these two cases are different. The last two types are the high-frequency excitation signal form (Fig. 3c,d), which more realistically depict the pavement. The equations describing these excitation signals are shown as follows:

46 $$ z_{r} \left( t \right) = z_{0} \sin \left( {2\pi ft + \varphi } \right), $$

47 $$ z_{r} \left( t \right) = 2\pi \int\limits_{0}^{t} {\left[ { - fz_{r} \left( \tau \right) + \sqrt {Gv\omega \left( \tau \right)} } \right]d\tau } . $$

The amplitude and frequency of the oscillation will be changed by the investigation case. The output of the problem is values such as displacement of the sprung mass, acceleration of the sprung mass, dynamic load, and voltage of the controller. These values are calculated according to their maximum value and average value.

The maximum value of the output signal:

48 $$ y_{max} = Max\left( {y_{i} } \right). $$

The average value of the output signal:

49 $$ y_{average} = \sqrt {\frac{1}{T}\int\limits_{0}^{T} {f^{2} \left( {y_{i} } \right)dy} } . $$

The parameters used for the simulation are given in Table 1. These parameters are obtained from CARSIM^®^ software and in Ref.^44^.

**Table 1:** Vehicle specification.

Symbol	Description	Value	Unit
m_s_	Sprung mass	570	kg
m_u_	Unsprung mass	62	kg
C	Damper coefficient	3850	Nsm^−1^
K	Spring coefficient	43,500	Nm^−1^
K_T_	Tire coefficient	182,000	Nm^−1^
κ_1_	Actuator coefficient	539,561	N^3/2^ kg^−1/2^ m^−1/2^ V^−1^
κ_2_	Actuator coefficient	1	s^−1^
κ_3_	Actuator coefficient	5,512,500	Nm^−1^

### Results and discussions

**Figure 4:**
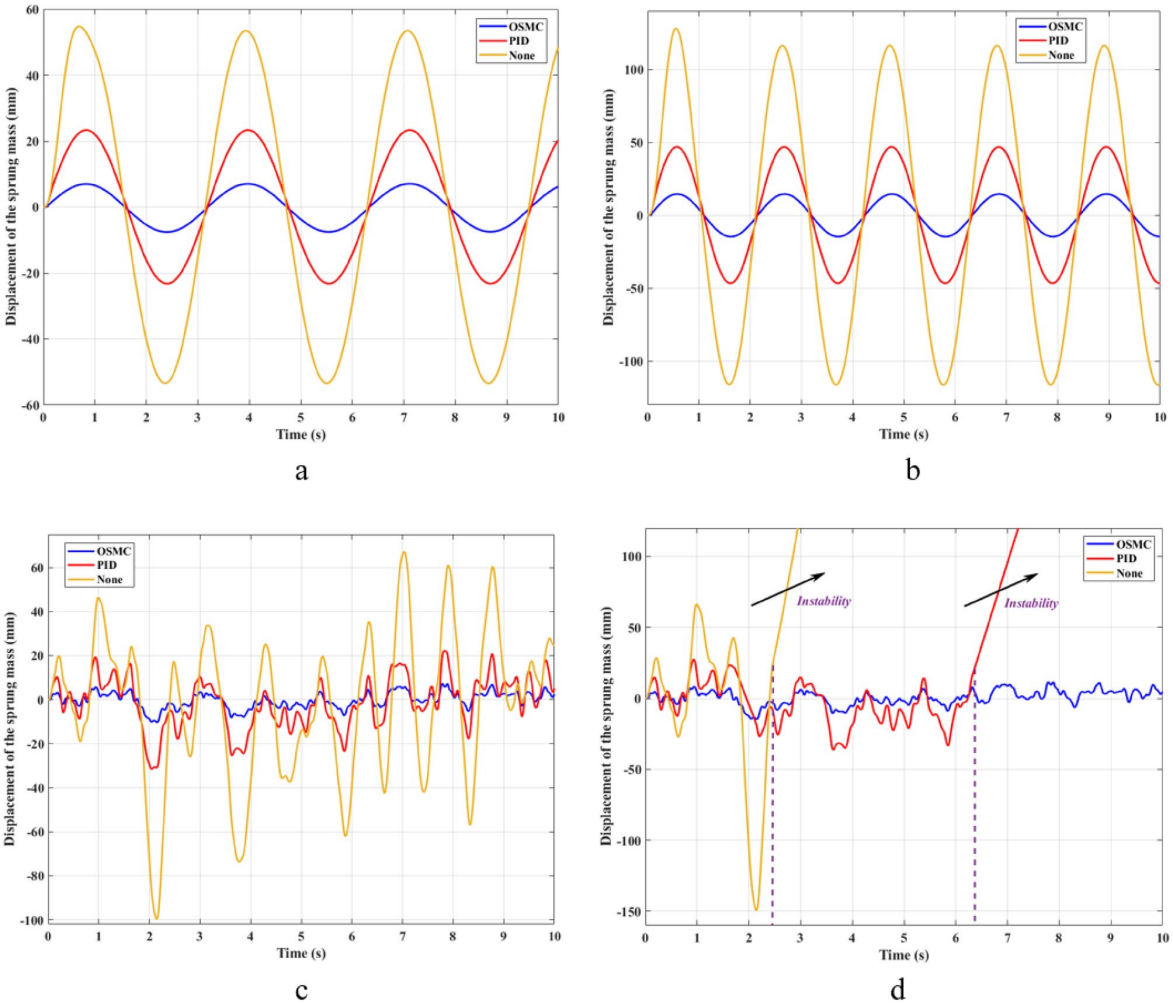
Displacement of the sprung mass ((**a**) Case 1; (**b**) Case 2; (**c**) Case 3; (**d**) Case 4).

The results of the calculation and simulation are given in the graphs below. Figure 4 shows the displacement of the vehicle body over time. According to this result, the value of the displacement of the sprung mass is minimized when the OSMC algorithm for the active suspension system is used. On the contrary, its value can be maximized if the vehicle is only equipped with a mechanical suspension system. In the first case (a), its maximum value reaches 54.76 (mm), 23.32 (mm), and 7.58 (mm) respectively, corresponding to the three investigation situations. Besides, the average value calculated according to the RMS criterion is 22.40 (mm), 9.72 (mm), and 3.04 (mm) respectively. In the second case (b), the amplitude and frequency of the oscillation have been increased. As a result, the displacement values of the vehicle body are also increased. The values obtained in the second case reach 128.05 (mm), 46.79 (mm), and 14.76 (mm) for the maximum values, respectively; 82.32 (mm), 32.97 (mm), and 10.30 (mm) for mean values. These are only cyclic excitations, so the variation of values is also cyclical. Compared with the results in Ref.^29^, which applied the SMC algorithm to the suspension, the value of vehicle body displacement obtained from this paper is much smaller. Besides, the system’s stability is also better when using the OSMC algorithm instead of the SMC algorithm.

In the other two cases, random stimulation was used. Therefore, the vehicle’s oscillation will also change continuously over time. In the third case (c), body vibration can reach 100.83 (mm) if the vehicle is not equipped with an active suspension system. In contrast, the maximum value of the displacement of the sprung mass is only about 31.90 (mm) and 10.57 (mm) if the vehicle uses an active suspension system with a hydraulic actuator. The average value of oscillations in these two situations is only 34.05% and 10.60% respectively, compared with the previous situation. Vehicle instability occurs in the last case (d). According to the results of Fig. 4d, the wheel will be lifted off the road at a time t = 2.5 (s), corresponding to the situation of the vehicle using only the passive suspension system. Besides, instability can also occur even when the vehicle has used the active suspension system. If the PID algorithm for the SISO system is applied, this instability still occurs at time t = 6.4 (s). Meanwhile, the OSMC algorithm still helps to ensure the stable performance of the vehicle during movement. The acceleration values obtained using the OSMC algorithm are all smaller than the conventional SMC algorithm^28,29^. Therefore, the smoothness of the car is better.

**Figure 5:**
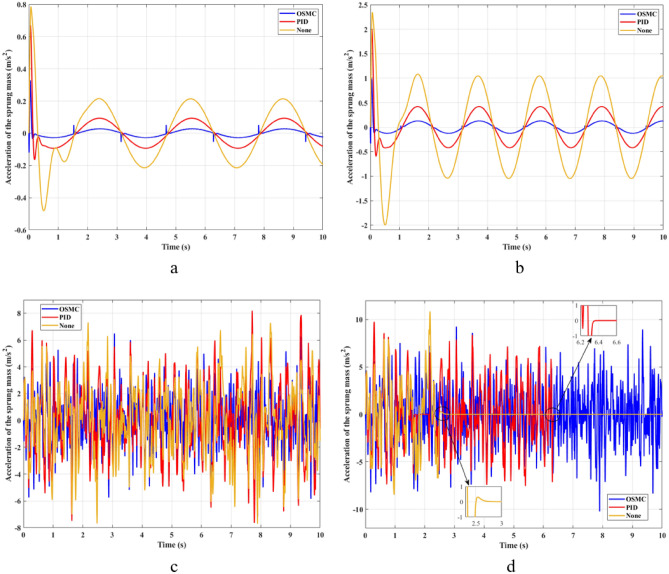
Acceleration of the sprung mass ((**a**) Case 1; (**b**) Case 2; (**c**) Case 3; (**d**) Case 4).

The acceleration of the sprung mass is considered to evaluate the smoothness when traveling on the road. The change in the acceleration of the sprung mass is shown in Fig. 5. In the first two cases, the acceleration is maximal at the first instant. Their maximum values corresponding to the three situations examined are: 0.79 (m/s^2^), 0.67 (m/s^2^), and 0.33 (m/s^2^) for the first case; 0.82 (m/s^2^), 0.34 (m/s^2^), and 0.11 (m/s^2^) for the second case. After the first phase of the oscillation, the acceleration value of the vehicle body will gradually decrease and change periodically with time. Their average values are, respectively, 0.29 (m/s^2^), 0.30 (m/s^2^), and 0.07 (m/s^2^) for the first case; 0.82 (m/s^2^), 0.34 (m/s^2^), and 0.11 (m/s^2^) for the next case. In the latter two cases, random oscillation will cause the vehicle's acceleration to change more. In the third case (c), the value of the acceleration varies continuously with time. If the vehicle is only equipped with a mechanical suspension system, the maximum and average values of acceleration can be up to 7.79 (m/s^2^) and 2.65 (m/s^2^). The value of acceleration when the vehicle uses an active suspension system with a PID controller is even greater than the situation where the vehicle only has a conventional passive suspension system. Its values can range between 8.28 and 2.73 (m/s^2^). This can increase the vehicle’s loss of comfort. However, the ride comfort can be improved when the OSMC algorithm is used for the suspension system. This improvement is not much, only 7.22 (m/s^2^), and 2.04 (m/s^2^). In the last situation, the performance of the OSMC controller is fully utilized. The vehicle still oscillates stably when the nonlinear control algorithm is used, while wheel separation has occurred in the other two situations.

**Figure 6:**
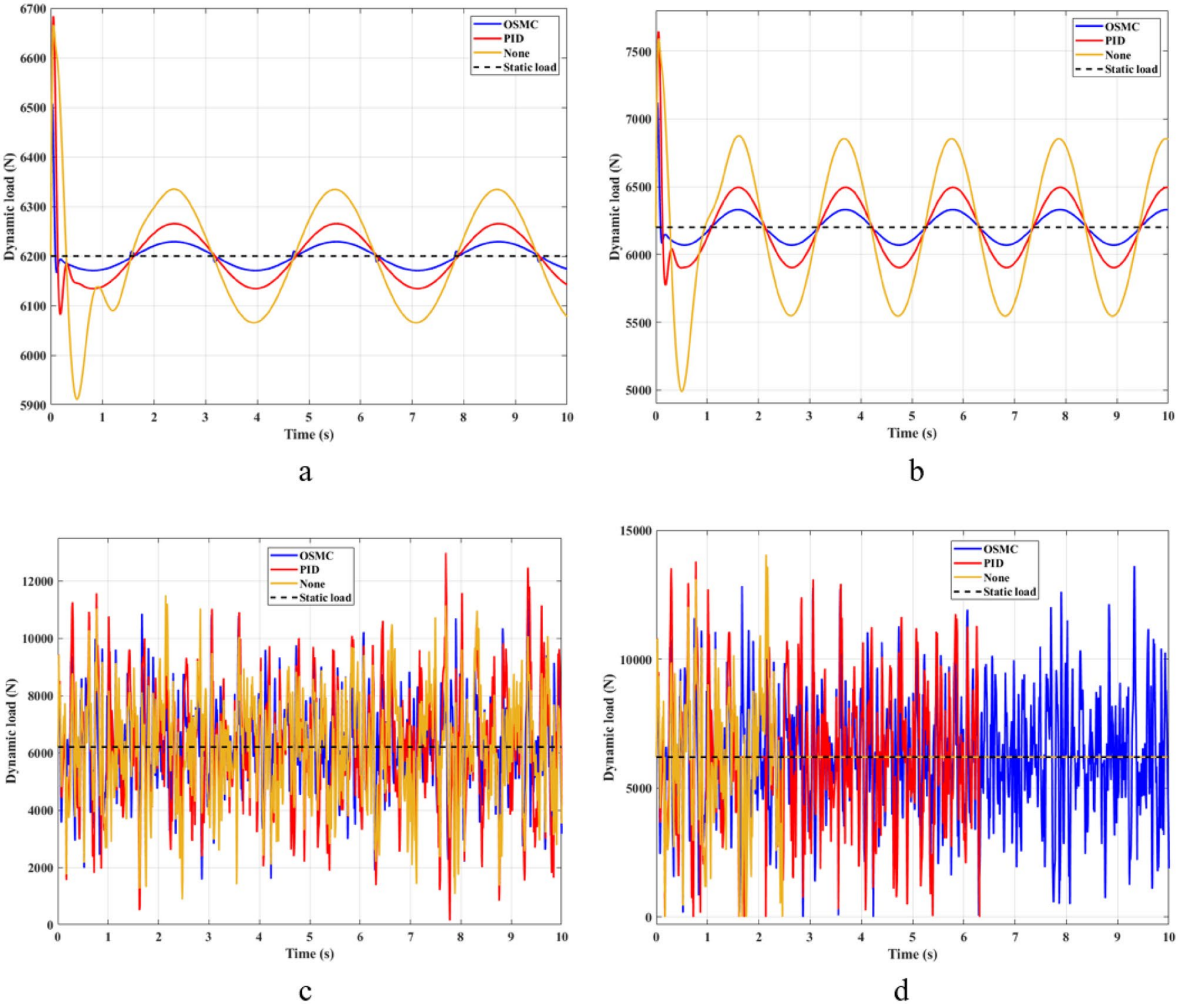
Dynamic load ((**a**) Case 1; (**b**) Case 2; (**c**) Case 3; (**d**) Case 4).

**Figure 7:**
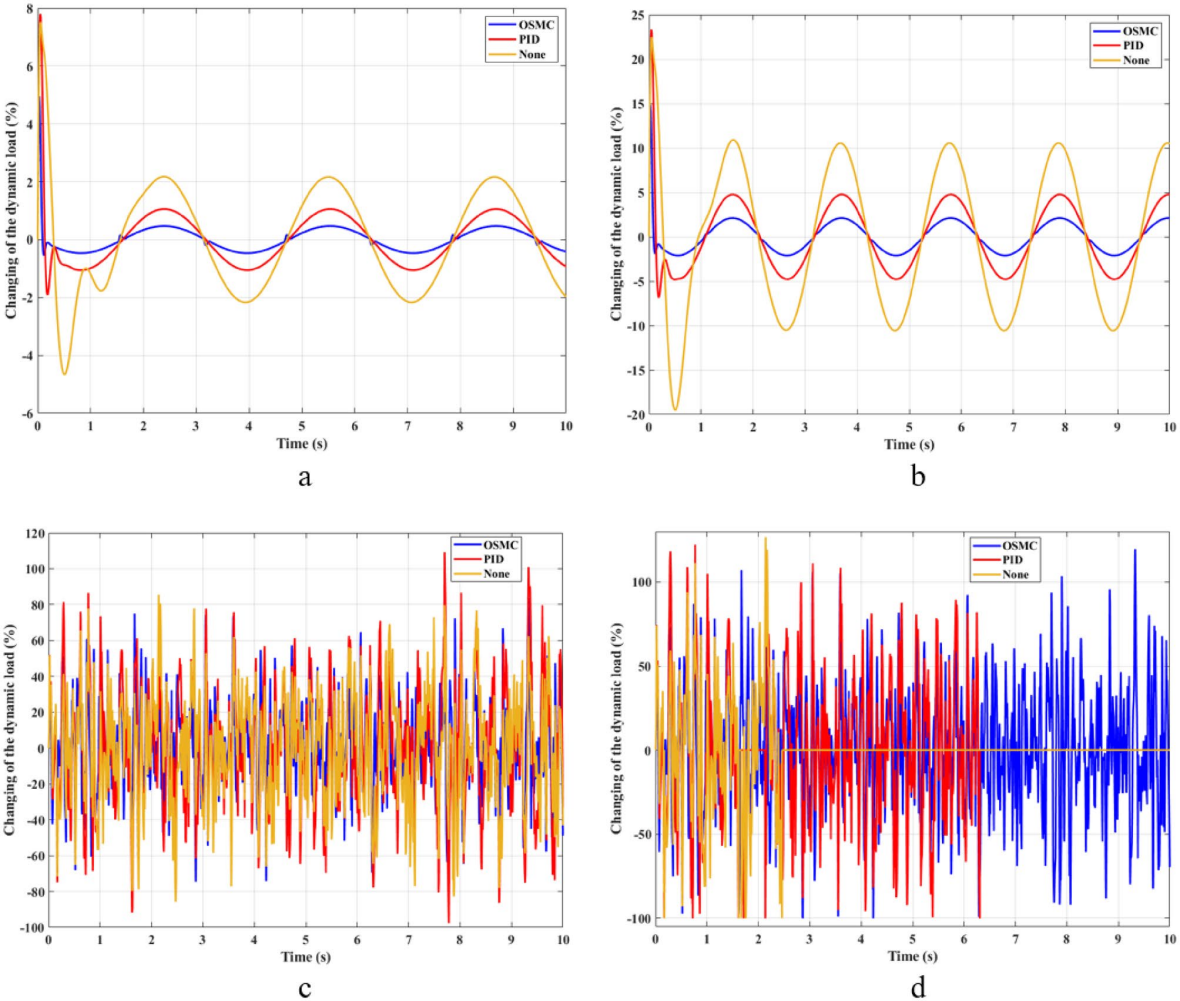
Changing of the dynamic load ((**a**) Case 1; (**b**) Case 2; (**c**) Case 3; (**d**) Case 4).

The interaction between the wheel and the road surface is expressed through the change of the load at the wheel, i.e., the dynamic load (Fig. 6). With cyclic oscillation, the load change is small (Fig. 7), and the wheel stays on the road. With random oscillations, the variation of the load is much larger. In some cases, the dynamic load approaches zero, which means the wheel is detached from the road surface. At this point, instability will occur. If the wheel separation occurs only for a small period, the instability will not be large. Conversely, if the load variation continues to increase, the wheels will no longer be able to interact with the road surface. To ensure the stability of the vehicle when traveling on the road, the change in the dynamic load should not exceed 100%.

**Figure 8:**
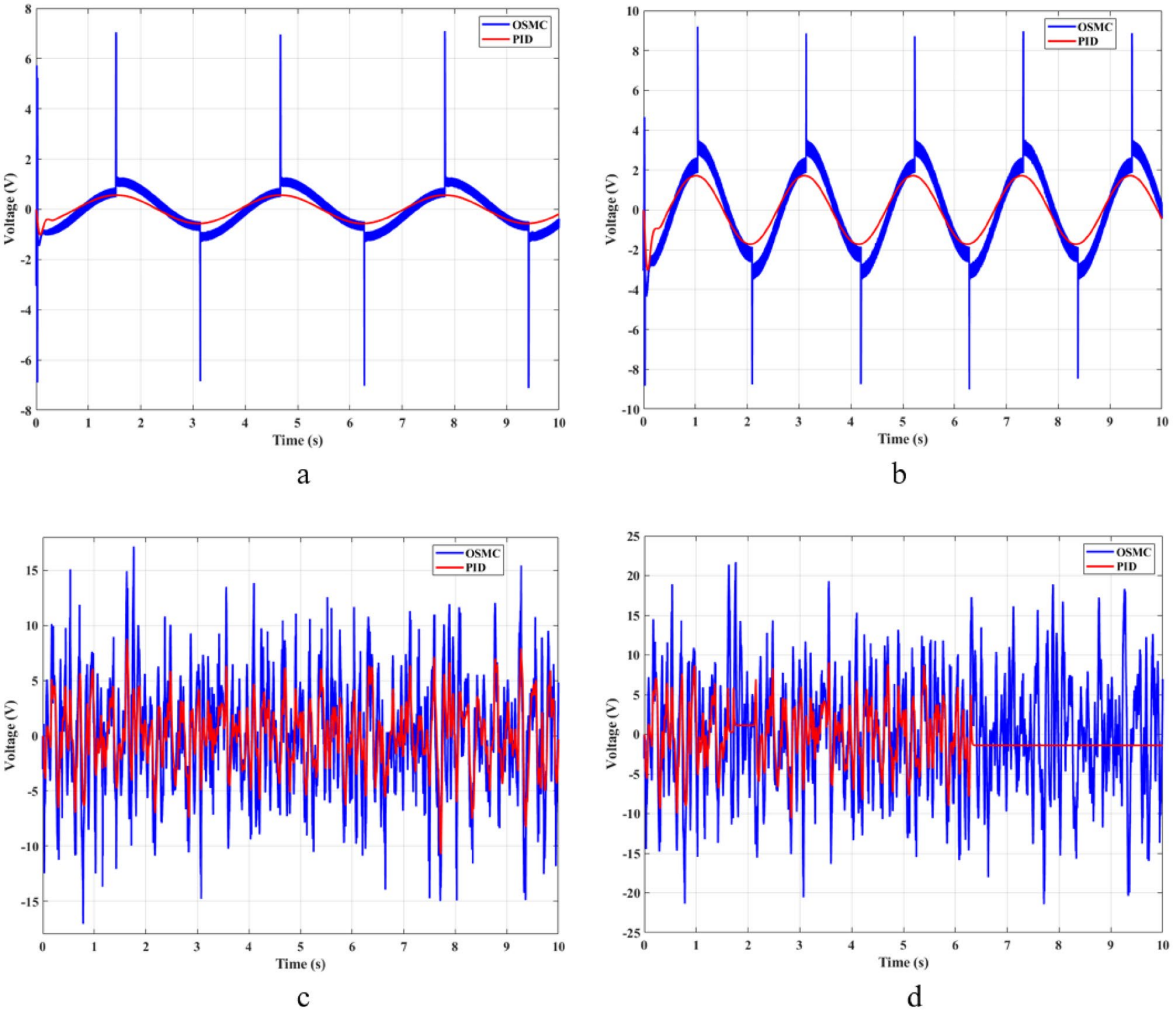
Voltage ((**a**) Case 1; (**b**) Case 2; (**c**) Case 3; (**d**) Case 4).

The impact force generated by the hydraulic actuator is dependent on the voltage supplied by the controller. Compared with traditional linear control algorithms, the OSMC algorithm will cause “chattering” (Fig. 8). This phenomenon will cause the control signal to vibrate continuously. In some cases, this phenomenon is harmful. However, the influence of the “chattering” phenomenon on the suspension control performed in this article is not large. Besides, the response time of the voltage signal when using the OSMC controller is also better than that of the PID controller. This makes the system more stable and efficient.

The results of the simulation are summarized in Tables 2, 3, 4, and 5.

**Table 2:** Results of the case 1.

	OSMC		PID		None	
	Value (mm)	Percent (%)	Value (mm)	Percent (%)	Value (mm)	Percent (%)
**Displacement of the sprung mass**						
Maximum	7.58	13.84	23.32	42.59	54.76	100.00
Average	3.04	13.57	9.72	43.39	22.40	100.00
**Acceleration of the sprung mass**						
Maximum	0.33	41.77	0.67	84.81	0.79	100.00
Average	0.07	24.14	0.30	103.45	0.29	100.00

**Table 3:** Results of the case 2.

	OSMC		PID		None	
	Value (mm)	Percent (%)	Value (mm)	Percent (%)	Value (mm)	Percent (%)
**Displacement of the sprung mass**						
Maximum	14.76	11.53	46.79	36.54	128.05	100.00
Average	10.30	12.51	32.97	40.05	82.32	100.00
**Acceleration of the sprung mass**						
Maximum	0.99	42.13	2.01	85.53	2.35	100.00
Average	0.11	13.41	0.34	41.46	0.82	100.00

**Table 4:** Results of the case 3.

	OSMC		PID		None	
	Value (mm)	Percent (%)	Value (mm)	Percent (%)	Value (mm)	Percent (%)
**Displacement of the sprung mass**						
Maximum	10.57	10.48	31.90	31.64	100.83	100.00
Average	3.58	10.60	11.50	34.05	33.77	100.00
**Acceleration of the sprung mass**						
Maximum	7.22	92.68	8.28	106.29	7.79	100.00
Average	2.04	76.98	2.73	103.02	2.65	100.00

**Table 5:**
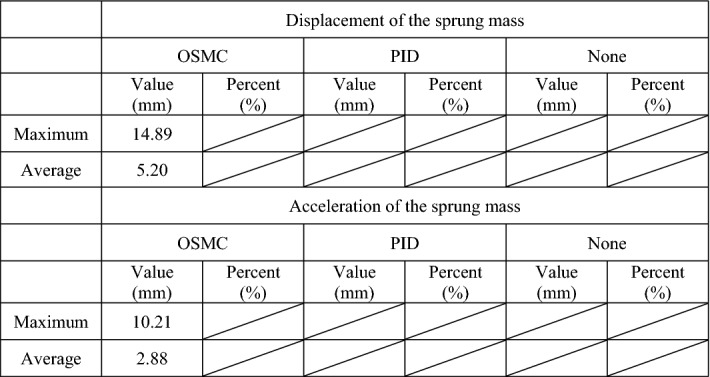
Results of the case 4.

## Conclusions

Road surface roughness is the cause of the vehicle’s vibrations when traveling on the road. This oscillation can affect passengers and cargo. The active suspension system is used to reduce these oscillations. This article focuses on the study and application of the nonlinear control algorithm for the active suspension system. In this study, a quarter-dynamics model with a hydraulic actuator is included. The calculations and simulations take place in the MATLAB-Simulink environment. There are four simulated cases corresponding to the four pavement excitation types. In these cases, three situations are considered.

According to the simulation results, the maximum values and the average values of the vehicle body are very large when the vehicle uses only the passive suspension system. If an active suspension system with a PID algorithm is used, the vehicle’s vibrations can be slightly improved. However, this improvement is not much. Even the vehicle’s vibration is stronger in some cases. Vehicle oscillation is improved when the OSMC algorithm is used to control the active suspension system. The above values are all at their stable level when this algorithm is applied to the simulation process. Besides, the wheels always interact well with the road surface. The phenomenon of wheel separation can be more radically limited.

Compared with traditional control algorithms, the OSMC algorithm is much more complex. The effectiveness of this controller depends on the design of the sliding surface and the selection of state vectors. The “chattering” phenomenon still occurs on the output signal of the controller. However, its influence is very small. To improve the performance and stability of the controller, the OSMC algorithm can be combined with the Fuzzy and PID algorithms. This content will be continued in the next article.

## Data Availability

The data used to support this research are included within this article.

## List of symbols

F_A_: Force of the hydraulic actuator, N
F_C_: Force of the damper, N
F_K_: Force of the spring, N
F_KT_: Force of the tire, N
F_i_: Force of the inertia, N
z_r_: Roughness on the road, m
z_s_: Displacement of the sprung mass, m
z_u_: Displacement of the unsprung mass, m

## Abbreviations

GSA: Gravitational search algorithm
LQR: Linear quadratic regulator
MIMO: Multi input–multi output
OSMC: Optimization–sliding mode control
PID: Proportional–derivative–integral
PSO: Particle swarm optimization
SISO: Single input–single output
SMC: Sliding mode control
